# RNA-Seq Reveals that Light and Darkness Are Different Stimuli in Freshwater Heterotrophic Actinobacteria

**DOI:** 10.3389/fmicb.2021.739005

**Published:** 2021-11-01

**Authors:** Priscilla P. Hempel, Jessica L. Keffer, Julia A. Maresca

**Affiliations:** ^1^Center for Bioinformatics and Computational Biology, University of Delaware, Newark, DE, United States; ^2^Department of Civil and Environmental Engineering, University of Delaware, Newark, DE, United States

**Keywords:** freshwater, light-dark cycle, Actinobacteria, transcriptome, competence, cryptochrome

## Abstract

Light is a ubiquitous source of both energy and information in surface environments, and regulates gene expression not only in photosynthetic microorganisms, but in a broad range of photoheterotrophic and heterotrophic microbes as well. Actinobacteria are keystone species in surface freshwater environments, where the ability to sense light could allow them to coordinate periods of nutrient uptake and metabolic activity with primary production. The model freshwater Actinobacteria *Rhodoluna* (*R.*) *lacicola* strain MWH-Ta8 and *Aurantimicrobium* (*A.*) *photophilum* strain MWH-Mo1 grow faster in the light than in the dark, but do not use light energy to support growth. Here, we characterize transcription throughout a light-dark cycle in *R. lacicola* and *A. photophilum*. In both species, some genes encoding carbohydrate metabolism and storage are upregulated in the light. However, expression of genes of the TCA cycle is only coordinated with light availability in *R. lacicola.* In fact, the majority of genes that respond to light and darkness in these two species are different, even though their light-responsive phenotypes are similar. The ability to respond to light and darkness may be widespread in freshwater Actinobacteria, but the genetic networks controlled by these two stimuli may vary significantly.

## Introduction

Light is a ubiquitous resource in surface environments, and widely used by microbes. In fact, light-sensing proteins that control gene expression are common in photosynthetic microbes, photoheterotrophs, and non-phototrophic heterotrophs (Eelderink-Chen et al., 2021). The organisms that do not use light energy for carbon fixation can use it for supplementary energy (Calisto et al., 2021), phototaxis (Wilde and Mullineaux, 2017), or to entrain circadian rhythms (Sartor et al., 2019). In non-phototrophic bacteria, light regulates multiple biological processes, including motility, pigment production, and stress responses (Burchard and Dworkin, 1966; Bhaya, 2004; Ziegelhoffer and Donohue, 2009). In photoheterotrophs, light often regulates expression of the photosystems, but may also regulate expression of the biosynthetic pathways of pigments or other photoactive cofactors, electron transport pathways, and the metabolic pathways that intersect with those (Frühwirth et al., 2012; Kumka et al., 2017; Navid et al., 2019). These pathways can also be regulated by oxygen tension, nutrient availability, and other environmental factors, resulting in complex regulatory networks in photoheterotrophs.

In illuminated freshwater environments, Actinobacteria in the Microbacteriaceae family are ubiquitous and abundant keystone species which mediate fluxes of organic carbon and nitrogen, reduced sulfur, and vitamins (Eiler et al., 2012; Garcia et al., 2018; Linz et al., 2018). The freshwater clades have in common small genomes (<2 Mbp) with low GC content compared to other Actinobacteria (∼50% GC), and a variety of auxotrophies (Neuenschwander et al., 2018). Both environmental metagenomic analyses and laboratory studies suggest that many members of these clades may be photoheterotrophs: actinorhodopsins and heliorhodopsins are common in their genomes, and both rhodopsin types can act as light-activated proton pumps (Ghai et al., 2014; Keffer et al., 2015; Dwulit-Smith et al., 2018; Pushkarev et al., 2018; Maresca et al., 2019).

Our previous work demonstrated that two species of these freshwater Actinobacteria, *Rhodoluna* (*R.*) *lacicola* strain MWH-Ta8 and *Aurantimicrobium (A.) photophilum* strain MWH-Mo1, grow faster in blue light than in the dark, even though neither has a functional rhodopsin under laboratory conditions (Maresca et al., 2019). Both species are free-living heterotrophs Hahn et al. (2021, 2014) Their growth rate phenotype strongly implies either that the cells have different activities in these two conditions, or that metabolic rates increase in the light. RNA-seq analysis of gene expression in stationary-phase cells grown in constant light or darkness further indicated that cells of both strains grown in constant light had higher expression of carbohydrate transport and metabolism pathways, while cells grown in constant darkness expressed more genes related to protein production and oxidative stress (Maresca et al., 2019). These transcriptional differences suggest that different metabolic pathways are active in light and darkness, and that they are transcriptionally regulated in response to light.

To begin to characterize the genetic and regulatory networks underlying the light-enhanced growth phenotype, we grew *R. lacicola* and *A. photophilum* in a 12-h light/12-h dark cycle and sequenced RNA from samples collected throughout the cycle. *R. lacicola* is representative of the Luna-1 clade of freshwater Actinobacteria, and *A. photophilum* is representative of the Luna-2 clade (Newton et al., 2011). The *R. lacicola* genome is smaller (∼1.4 Mbp as compared to ∼1.8 Mbp), and the two genomes share 879 genes, representing 65% of the *R. lacicola* genome and 50% of the *A. photophilum* genome (Supplementary Figure 1; Maresca et al., 2019). We initially predicted that the transcriptional responses of the two strains to light and darkness would be similar, because they belong to the same family (Microbacteriaceae), their genomes are so similar, and their light-enhanced growth phenotypes are similar. Here, we show that light and darkness alter transcription of distinct suites of genes in *R. lacicola* and *A. photophilum*, that approximately half of the genes in both are regulated in response to light or darkness, and that although the growth rate phenotypes of these strains are similar, their transcriptional responses to light and darkness are quite different.

## Materials and Methods

### Strains and Growth Conditions

*A. photophilum* strain MWH-Mo1 and *Rhodoluna lacicola* strain MWH-Ta8 were grown as described previously in 0.3% NSY medium (Hahn et al., 2004) at room temperature (Maresca et al., 2019). For the light/dark cycling experiment, for each sample to be collected, cells were diluted into 50 mL of fresh media (the starting OD_600__nm_ was 0.003 for *R. lacicola* and 0.008 for *A. photophilum*) and placed on an orbital shaker in a dark room. White light was provided by four 13-watt compact fluorescent bulbs (each providing ∼30–40 μmol photons m^–2^ s^–1^) arranged approximately 30 cm above the cultures, on a 12-h ON/12-h OFF cycle. The cultures acclimated to the light/dark cycle for 24 h before sampling began. The first sample was taken in the dark, ∼5 min before the light turned on (t_0_). Then, samples were taken 15 min (t_1_, 0.25 h), 1 h (t_2_), and 6 h (t_3_) after the light turned on, and 15 min (t_4_, 12.25 h), 1 h (t_5_, 13 h), and 6 h (t_6_, 18 h) after the light turned off. For each time point and each strain, the entire culture (50 mL) was centrifuged at 4,500 × *g* for 30 min at room temperature. The supernatant was removed and the cell pellet was resuspended in 1 mL RNAlater (Invitrogen) and stored at −20^o^C until processing. Biological replicates were obtained by repeating the experiment 3 times for a total of 4 replicates, ensuring that the initial concentration of cells remained the same.

### RNA Extraction and cDNA Library Preparation

The archived cell suspensions were separated into four aliquots, and RNA was extracted from one aliquot of each *R. lacicola* sample and from two pooled aliquots of *A. photophilum* samples. The remainder of the material was archived at −80^*o*^C.

Total RNA from *R. lacicola* was extracted as described previously (Maresca et al., 2019). RNA yield from *A. photophilum* was too low when cells were lysed with the enzymatic digestion protocol alone, so cells were lysed by sonication. Cells were centrifuged to remove the RNA*later* solution, then resuspended in lysis buffer (30 mM Tris-HCl, 1 mM EDTA, pH 8.0) with lysozyme (15 mg mL^–1^) and proteinase K (2 mg mL^–1^). Immediately prior to sonication, Buffer RLT (Qiagen) was added. Cells were then lysed using a Branson Sonifier 450 equipped with a microtip (10 cycles, 60% duty cycle). After sonication, 500 μL 100% ethanol was added, and purification of total RNA continued using the RNeasy Mini Kit (Qiagen 74104) as described previously (Maresca et al., 2019).

For both strains, residual DNA was removed by treatment with TURBO DNase (Ambion AM1907) and RNA quality was assessed using an AATI fragment analyzer. RNA was concentrated using the RNeasy MinElute Cleanup kit (Qiagen 74204) and quantified using the Qubit RNA BR assay kit (Invitrogen Q10210). The samples sent to the Joint Genome Institute (JGI) for library preparation and sequencing were 25 μL each, containing 25–359 ng RNA μL^–1^.

### Library Preparation and Illumina Sequencing

Library preparation and sequencing was carried out at the JGI. For each sample, rRNA was removed from 100 ng of total RNA using the Ribo-Zero Bacterial rRNA Removal Kit (Illumina). Stranded cDNA libraries were generated using the Illumina Truseq Stranded mRNA Library Prep Kit. The rRNA-depleted RNA was fragmented and reverse transcribed using random hexamers and SSII (Invitrogen) followed by second strand synthesis. The fragmented cDNA was treated with end-repair, A-tailing, adapter ligation, and 10 cycles of PCR. The prepared libraries were quantified using KAPA Biosystem’s next-generation sequencing library qPCR kit and run on a Roche LightCycler 480 real-time PCR instrument. Sequencing of the flowcell was performed on the Illumina HiSeq2500 sequencer using HiSeq TruSeq SBS sequencing kits, v4, following a 2 × 150 nt indexed run recipe.

### Read Preprocessing and Filtering

Standard pre-processing and filtering were done at the JGI using standard JGI pipelines. Raw fastq file reads were filtered and processed using BBDuk (Bushnell, 2014) and its microbial transcriptome filtering options to remove calibration reads, trim reads that contained adapter sequences, trim reads where quality drops to 0, and remove any reads from contaminants. Raw reads were evaluated for artifact sequence by kmer matching (kmer = 25), allowing 1 mismatch, and detected artifacts were trimmed from the 3′ end of the reads. Reads that contained 1 or more ‘N’ bases, had an average quality score across the read less than 10 or had a minimum length ≤ 51 bp or 33% of the full read length, were removed. Reads mapped with BBMap (Bushnell, 2014) to masked human, cat, dog and mouse references at 93% identity were removed. Reads aligned to common microbial contaminants and rRNA were also removed.

### Read Mapping, Counting, and Replicate Analysis

The raw reads from each library were aligned to the reference genomes using BBMap (Bushnell, 2014) with only unique mappings allowed. If a read mapped to more than one location, it was ignored. To generate the raw gene counts, featureCounts was used (Liao et al., 2014). Counts refer to paired reads, so in cases where both reads aligned to the same feature in the reference genome, it was represented as a count of 1 in the gene counts table. These raw gene counts were normalized to gene length and library size. The normalized counts were then used to evaluate the level of correlation between biological samples using Pearson’s correlation, but these normalized counts were not used for other downstream analyses. Four of the 28 *A. photophilum* samples (one each from t_0_, t_1_, t_2_, and t_5_) were excluded from downstream analyses because they did not correlate well with their replicate group, as determined by a Pearson correlation calculation.

### Principal Component Analysis

To evaluate the similarity of the replicate samples, the plotPCA function of the BiocGenerics R package version 0.34.0 (Huber et al., 2015) was used for principal component analysis. Raw count data was regularized logarithm (rlog) transformed via the rlog function of the DESeq2 R package, version 1.28.1 (Love et al., 2014) and used as input for the PCA analysis. The rlog transformation was applied to the count data to normalize with respect to library size and to decrease differences between samples for genes with low counts. In *A. photophilum*, this analysis showed that 4 samples from different treatment groups separated from all other samples. These four samples (one each from t_3_, t_4_, t_5_, and t_6_) were located in the same column of the sequencing plate, and data from these samples was also excluded from further analysis. With the 4 samples removed in the earlier stage, this resulted in exclusion of a total of 8 samples, leaving 20 samples from *A. photophilum* for downstream analysis.

### Differential Expression Analysis

For differential expression analysis, raw gene counts were input to the DESeq2 R package, version 1.28.1 (Love et al., 2014), which fits a negative binomial model for abundance of each transcript. A likelihood ratio test (LRT) with a full model design of ∼ condition (condition = sampling time) was used to identify genes for which time explains a significant amount of variation in the data. The full model was compared to a reduced model in which sampling time was not included as a variable. The p-values were adjusted using the Benjamini and Hochberg (BH) method in DESeq2, and the significance threshold for differential gene expression was an adjusted *p*-value ≤ 0.015.

### Gene Expression Pattern Clustering

To group genes by expression patterns throughout the time series, rlog-transformed count values of differentially expressed genes were input to the degPatterns function of the DEGreport R package, version 1.24.1 (Pantano, 2017). The degPatterns function with the ConsensusCluster parameter set to false applies the DIANA (DIvisive ANAlysis) hierarchical clustering-based approach to generate clusters of genes with similar expression patterns. Each replicate was input as an individual sample. All clusters with at least two genes were included in the output. The DIANA hierarchical clustering method provides the divisive coefficient which measures the strength of the cluster structure on a scale from zero to one, with a higher value indicating stronger cluster structure.

### Gene Ontology Enrichment

Gene Ontology (GO) annotations were assigned to identify functions in gene clusters according the GO database, which is the key functional classification of the National Center for Biotechnology Information (NCBI) (Gene Ontology Consortium, 2004). Protein sequences from both species were annotated with the Blast2GO (version 5.2.5) suite of tools (Götz et al., 2008), using BLASTP against the non-redundant protein database with an E-value cutoff of 1 × 10^–3^. The GO-slim function was applied to generate general GO-slim terms to obtain an overview of the biological processes, cellular locations, and molecular functions of predicted proteins. Gene groups were merged based on their mean expression pattern.

To determine enrichment of a GO-slim term in a merged gene group, odds ratios were calculated as the ratios of representation of a GO term within a merged group to the representation of the same term in the genome ([A/B]/[C/D], where A is the number of genes with a given GO-slim term in a group, B is the total number of genes assigned any GO-slim term in a group, C is the number of genes assigned the given GO-slim term in the genome, and D is the total number of genes assigned any GO-slim term in the genome.) The stats (version 4.0.4) R package was used to calculate the odds ratios and do a one-sided Fisher’s exact test (using the fisher.test function) to test for significance. Results were then filtered with criteria odds ratio > 1, a count in the merged group > 1, and a count in the whole genome ≥ 10.

Gene expression patterns were plotted as Z-score transformed transcript abundances. Z-score transformation standardizes data across sampling time points and allows for analysis that is independent of the raw count transcript abundance. Z-score were calculated by subtracting a gene’s mean transcript abundance across all timepoints from each sample, then dividing by the standard deviation of all timepoints. Z-scores for timepoint replicates were then averaged. The Z-score represents the number of standard deviations away from the mean transcript abundance across all timepoints. Rlog transformed transcript abundance values above the mean have a positive Z-score while rlog transformed transcript abundance values below the mean have a negative Z-score.

### Plotting

All plots were generated using the ggplot2 R package, version 3.3.3 (Wickham, 2009) and the patchwork R package, version 1.1.1 (Pedersen, 2020).

## Results

### Sequence Data

A total of 56 cDNA samples (2 strains × 7 time points × 4 replicates per time point) were sequenced by the JGI using an Illumina HiSeq system, paired-end 150-nt reads. For *R. lacicola*, we obtained a total of 718,789,878 raw reads (range 18,305,388–34,132,688 per sample) and 653,982,504 reads passed quality filtering (range 13,215,036–33,300,442 per sample), resulting in 326,991,252 total fragments (range 6,607,518–16,650,221) (Supplementary Table 1). 326,304,878 fragments were mapped to the reference genome (range 6,593,343–16,614,066) and 238,500,825 fragments were assigned to genes (range 4,488,560–12,174,032). Pearson correlations were calculated between all samples, and all replicates grouped together as expected.

For *A. photophilum*, we obtained 547,198,496 raw reads (17,947,434–34,160,484 per sample) and 455,067,112 reads (7,766,636–32,336,932 per sample) passed quality filtering, resulting in 227,533,556 total fragments (3,883,318–16,168,466 per sample) (Supplementary Table 2). A total of 222,502,773 fragments (3,800,635–15,523,899 per sample) were mapped to the reference genome and 167,358,807 (range 2,459,643–12,059,277) fragments were assigned to genes.

### Principal Component Analysis

**FIGURE 1:**
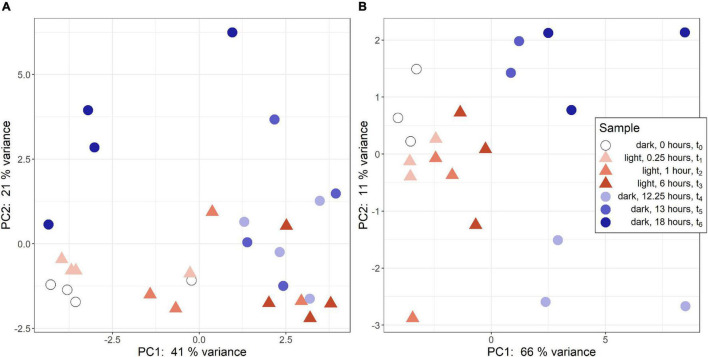
Principal component analysis (PCA) of RNA-seq samples. **(A)** PCA of rlog scaled count data from *R. lacicola*. The steady-state light and early dark timepoints are quite similar in *R. lacicola*, as are steady-state dark and early light timepoints. **(B)** PCA analysis of *A. photophilum* strain MWH-Mo1. Light and dark samples are clearly distinct from each other along PC1.

Regularized logarithmic (rlog) transformed count data was analyzed via a principal component analysis to compare treatment groups. In *R. lacicola*, PC1 best captures variance among light timepoints (Figure 1A). The first two dark timepoints, t_4_ and t_5_, group closely on both PC1 and PC2. However, PC1 captures variance between the first two dark timepoints and t_6_. Interestingly, t_6_ (6 h in the dark) groups closest to the first two light timepoints, possibly indicating a cyclical expression pattern. The replicates roughly group together and lie closest to their neighboring timepoints. In *A. photophilum*, PC1 captures the variance between light and dark timepoints (Figure 1B), indicating light and dark samples are quite different from each other, and PC2 captures variance between the early and late dark samples. Four samples from the *A. photophilum* dataset were removed due to the PCA analysis. These four samples were in the same column of the sequencing plate, from different timepoints (t_3_, t_4_, t_5_, t_6_) and grouped together along both PC1 and PC2, rather than with their timepoint replicates. Removing those samples left three replicates for each timepoint, except for t_5_, which had two replicates in downstream analyses. The two remaining t_5_ replicates cluster tightly together along PC1 and PC2 that collectively represent 76% of the variance. Of the top 100 genes with the highest loading values in PC1 and PC2, PC2 loading values were the most affected by removing the four samples. Nevertheless, PC2 captures variance between the early and late dark samples both before and after removing the outlying samples.

### Expression Group Analysis

**FIGURE 2:**
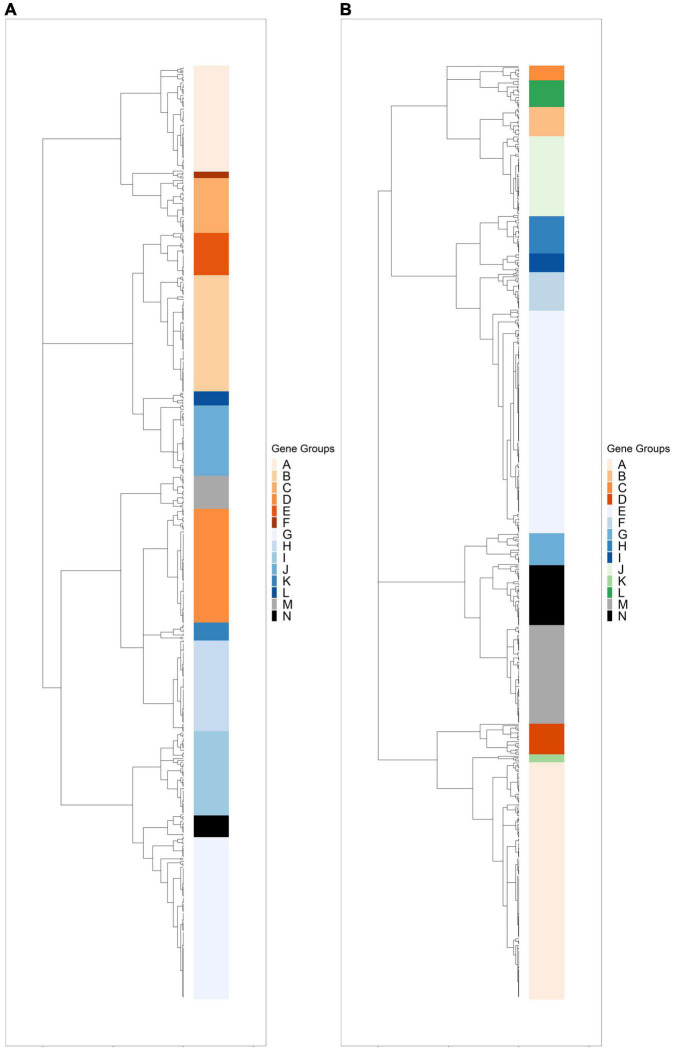
Hierarchical clustering of rlog scaled count data through the light-dark cycle. Differentially expressed genes were clustered based on expression patterns through time, and any cluster with fewer than two genes was excluded. **(A)** Dendrogram of hierarchically clustered differentially expressed genes in *R. lacicola*. Putative light-responsive genes whose transcript abundance increases during the first hour in the light and decreases between one hour and six hours in the dark are colored in orange shades. Putative light-responsive genes whose transcript abundance generally decreases during the first hour in the light and increases between one hour and six hours in the dark are colored in blue shades. The groups colored black and gray appear to respond to darkness. **(B)** Dendrogram of hierarchically clustered differentially expressed genes in *A. photophilum* strain MWH-Mo1. Colors indicate gene groups with similar expression profiles. Orange shades indicate genes whose expression increases within one hour in the light and decreases within one hour in the dark. Blue shades indicate genes whose expression decreases within one hour in the light and increases within one hour in the dark. Green indicates genes whose expression increases within one hour in the light and decreases within 15 min in the dark. Black and gray indicates genes whose expression decreases within one hour in the light and increases within 15 min in the dark.

**FIGURE 3:**
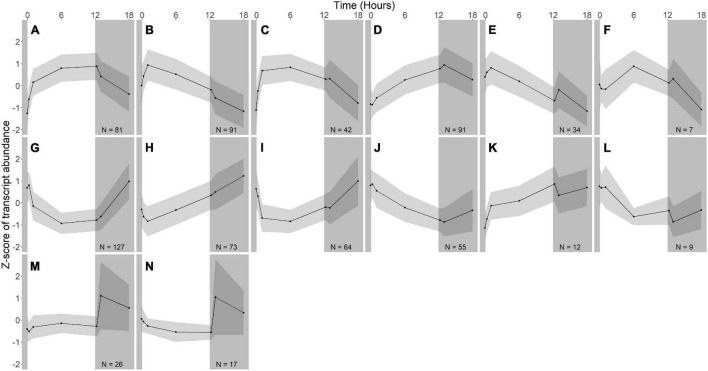
Gene expression groups in *R. lacicola*. Groups of genes with similar expression patterns were identified using hierarchical clustering. Means of the z-scores for all genes are plotted, with one standard deviation indicated by the gray shading. N indicates number of genes in each group. **(A–F)** Groups of light-responsive genes whose expression increases in the light and decreases in the dark. **(G–L)** Groups of light-responsive genes whose expression decreases in the light and increases in the dark. **(M,N)** Groups of genes whose expression is consistent until the light turns off, then increases sharply.

**FIGURE 4:**
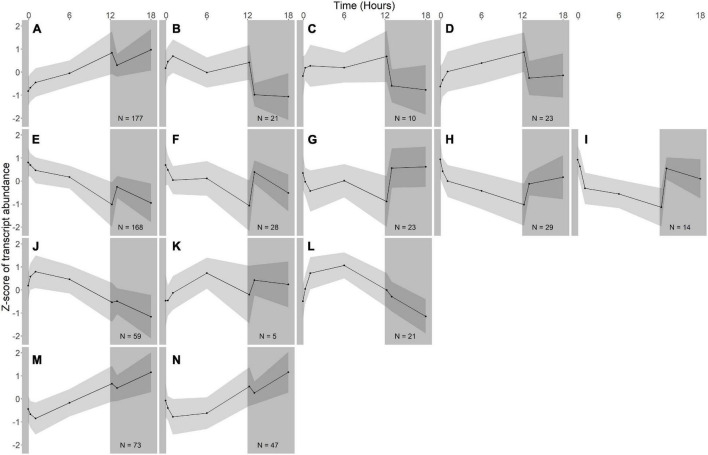
Gene expression groups in *A. photophilum* strain MWH-Mo1. Groups of genes with similar expression patterns were identified using hierarchical clustering. Means of the z-scores for all genes are plotted, with one standard deviation indicated by the gray shading. N indicates number of genes in each group. In this species, most genes respond more strongly to darkness than to light. **(A–D)** Groups of genes whose expression increases in the light and rapidly decreases in the dark. **(E–I)** Groups of genes whose expression decreases in the light and rapidly increases in the dark. **(J–L)** Groups of genes whose expression increases early during the light period, then decreases. **(M,N)** Groups of genes whose expression decreases early during the light period, then increases.

Hierarchical clustering was used to identify expression groups: groups of differentially expressed genes whose expression patterns were similar across all time points. The divisive coefficient, which indicates the strength of the cluster structure, is 0.998 for *R. lacicola* and 0.995 for *A. photophilum*, indicating very strong cluster structures in both species (maximum value = 1). Fourteen expression groups were identified in each strain (Figure 2). In *R. lacicola*, 729 genes representing ∼54% of all genes were clustered into three categories of expression groups: putative light-responsive groups (8), putative dark-responsive groups (2), and 4 groups which may respond to all changes in light availability (Figure 3). In *A. photophilum* strain MWH-Mo1, the transcripts of 40% of all genes appear to respond to light, darkness, or both (Figure 2B). In contrast to the patterns in *R. lacicola*, the largest change in transcript abundance occurs after the transition to darkness for nearly all of the genes (Figure 4).

In *R. lacicola*, the genes in the putative light-responsive groups either increase (Figures 3A–D) or decrease (Figures 3G–J) in expression level consistently during the light period, then decrease or increase during the dark time points, respectively. The genes in the putative dark-responsive group (∼3% of all genes) have fairly consistent expression levels throughout the light period, then increase sharply in the dark (Figures 3M,N). Four small groups of genes appear to change in transcript abundance in response to both light and darkness (Figures 3E,F,K,L). None of the genes unique to the *R. lacicola* genome appear in any of these groups.

**FIGURE 5:**
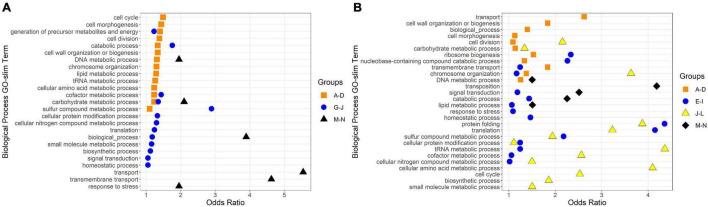
Biological Process GO-Slim Terms with significant enrichment in expression groups. Group letters correspond to those in Figures 3, 4. Odds ratios were calculated as the ratio of a term’s representation in a merged group to its representation in the whole genome; any odds ratios presented here have a ratio > 1, a count in the merged group > 1, and a count in the whole genome ≥ 10. **(A)** GO-slim terms enriched in *R. lacicola* expression groups. **(B)** GO-slim terms enriched in *A. photophilum* strain MWH-Mo1 expression groups.

**FIGURE 6:**
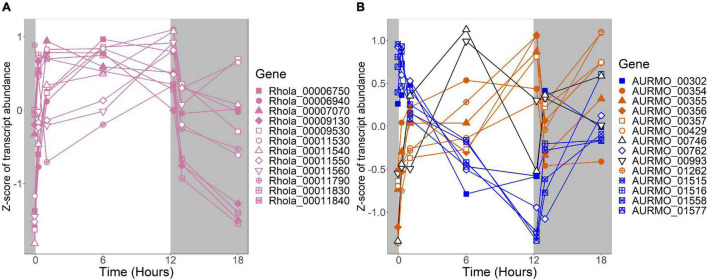
Expression of putative TCA cycle genes. Putative competence genes were identified by blasting the amino acid sequences of known components of the *E. coli* TCA cycle against the genomes of *R. lacicola* and *A. photophilum* strain MWH-Mo1. Expression is plotted as z-score of each transcript. **(A)** Putative TCA cycle genes in *R. lacicola* have higher transcript levels in the light. **(B)** Putative TCA cycle genes in *A. photophilum* strain MWH-Mo1.

**FIGURE 7:**
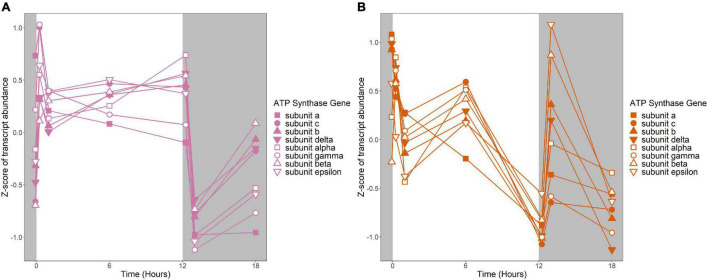
Expression of putative ATP synthase genes. Expression is plotted as z-score of each transcript. **(A)** Putative ATP synthase genes in *R. lacicola*. **(B)** Putative ATP synthase genes in *A. photophilum* strain MWH-Mo1.

To identify broad categories of functional genes in the expression groups, generic GO-slim terms (Götz et al., 2011) were mapped to the *R. lacicola* and *A. photophilum* genomes, which have similar content and distribution of GO-slim terms (Supplementary Figure 1). The biological process GO-slim terms present at a higher proportion in the expression groups than in the genome were then identified using an odds ratio calculation. In *R. lacicola*, putatively light-responsive groups whose expression increases in the light were enriched in genes associated with cell cycle/cell division, cell wall and morphogenesis processes, precursor metabolite production, DNA metabolic processes, and carbohydrate metabolic processes (Figure 5A), including two putative starch synthases (Supplementary Table 3) and most of the genes in the TCA cycle (Figure 6A). The putatively light-responsive groups of genes whose transcript abundance decreases during the light period, then increases in the dark (Figures 3G–J) were enriched in functions related to nitrogen metabolic processes, translation, and signal transduction. This group also includes the actinorhodopsin, DNA photolyase, and an uncharacterized cryptochrome-photolyase family protein (CPF2), as well as a peroxiredoxin (*Rhola_00006540*). A second peroxiredoxin, *Rhola_00011030*, does not fall into an expression group, but its expression clearly follows a light-dark cycle (Supplementary Figure 2A). Similarly, some components of the ATP synthase fall into group 3J, but all components have a similar expression pattern, and their transcription in *R. lacicola* is clearly stimulated by light and repressed in the dark (Figure 7A). Interestingly, sulfur metabolic processes are even more enriched in this group than in the other (Figure 5A and Supplementary Table 5). The group of potentially dark-responsive genes is enriched in transport, stress responses, carbohydrate metabolism, and DNA metabolism (Figure 5A and Supplementary Table 5).

**FIGURE 8:**
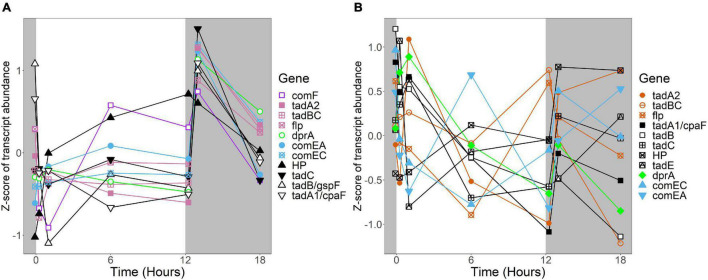
Expression of putative competence genes. Putative competence genes were identified by blasting the amino acid sequences of known components of the Com operon and *tad/flp* pilus from *Micrococcus luteus* against the genomes of *R. lacicola* and *A. photophilum* strain MWH-Mo1. In both strains, components of the putative *tad/flp* pilus are organized into two operons, and *comEC* and *comEA* form a separate putative operon. Expression is plotted as z-score of each transcript. **(A)** Putative competence genes in *R. lacicola*. **(B)** Putative competence genes in *A. photophilum* strain MWH-Mo1.

In *R. lacicola*, the relatively small group of putatively dark-regulated genes (Figures 3M,N) is enriched in transmembrane transport and carbohydrate metabolism (Figure 5A). These groups also include genes encoding DNA uptake (*comEC* and *dprA*) and putative components of a *tad/flp* pilus (two homologs of *tadA* and one *tadBC* homolog). Although not all of the genes putatively encoding components of the *tad/flp* pilus in *R. lacicola* fall into specific expression groups, expression of all except *comEA* increases sharply within 1 h of the light turning off (Figure 8A). Although the genome of *A. photophilum* strain MWH-Mo1 has homologs to all of these genes, none of them have expression patterns that imply that they respond to light or darkness or that they are coordinately regulated (Figure 8B and Supplementary Table 4).

Fourteen groups of genes with similar expression patterns were identified in *A. photophilum*, but the patterns are quite different from those observed in *R. lacicola* (Figure 4). The abundance of nearly all transcripts appears to change – sometimes dramatically – in response to darkness (Figure 4). The abundance of ∼80 transcripts in *A. photophilum* increases consistently in the light, then decreases (Figures 4J,L); these groups include functions related to carbohydrate metabolism, cell division, cell cycle, and biosynthesis (Figure 5B). In four *A. photophilum* expression groups, transcript abundance increases for the first hour in the light, and rapidly decreases between 15 min and 1 h in the dark (Figures 4A–D). These groups are enriched in functions related to transport, cell wall and morphogenesis, cell division, and carbohydrate metabolism (Figure 5B), and also include one of the two peroxiredoxins in the *A. photophilum* genome (*AURMO_00420*; Supplementary Figure 2B and Supplementary Table 4) and one of the two putative starch synthases (*AURMO_01094*; Supplementary Table 4). In five groups, transcript abundance decreases during the first hour in the light, stabilizes or increases through 6 h in the light, then increases between 15 min and 1 h in the dark (Figures 4E–I). These groups are enriched in functions including ribosome biogenesis, translation, protein folding, protein modification, sulfur metabolism, and signal transduction (Figure 5B and Supplementary Table 6), and the superoxide dismutase and phytoene synthase are in Group 4H (Supplementary Table 4). Expression patterns of the genes encoding components of the F_1_F_0_ ATP synthase, though they did not cluster with any of these groups, are most similar to the pattern of group 4F (Figure 7B). Last, two groups of genes decrease during the first hour in the light, then consistently increase throughout the rest of the experiment, with transient decreases in transcript abundance during the first hour in the dark (Figures 4M,N). These two groups are enriched in signal transduction, catabolism, lipid metabolism, and transposition (Figure 5B and Supplementary Table 6). Of the 120 genes in these two groups, 80 have no homolog in the *R. lacicola* genome.

In our prior work, genes related to carbohydrate metabolism were more highly expressed in the light than in the dark in both strains (Maresca et al., 2019). In this experiment, in *R. lacicola*, carbohydrate metabolic processes were enriched in all three merged expression groups. Groups A-D, in which transcript abundances increase in light and decrease in dark, contain genes related to gluconeogenesis, lipid metabolism, and synthesis of sugars. Groups G-J, in which transcript abundances decrease in light and decrease in dark, include genes related to glycolysis and the pentose phosphate pathway. Groups M-N, which drastically increase in the dark, contain 3 genes related to activating or isomerizing sugars for degradation (Supplementary Table 3). Genes in the TCA cycle fall into all three of these groups, and thus the pathway as a whole does not seem to be co-regulated (Figure 6B). In *A. photophilum*, only 13 carbohydrate metabolism genes clustered with expression groups. Carbohydrate metabolic processes were enriched in groups A-D and J-L, in which transcription increases for the first hour after the light turns on. Groups A-D include genes related to glycogen metabolism and glycogenesis. Groups J-L include 4 genes related to glycolysis, the pentose phosphate pathway, and a gene related to nucleotide-sugar biosynthesis.

### Expression of Putative Light Sensors

**FIGURE 9:**
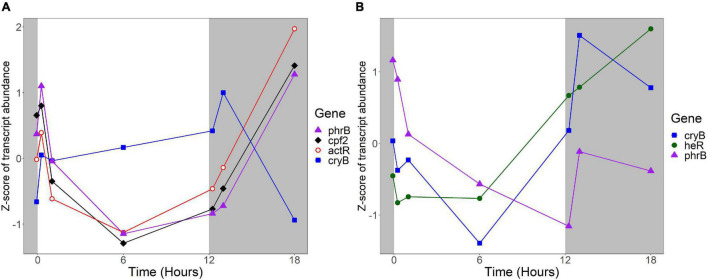
Expression of putative light sensors. Expression is plotted as z-score of each transcript. **(A)** Expression of potential light sensors in *R. lacicola*, which include an actinorhodopsin (*actR*), a DNA photolyase (*phrB*), a CryB-type cryptochrome (*cryB*), and another member of the cryptochrome-photolyase superfamily of proteins (*cpf2*). **(B)** Expression of potential light sensors in *A. photophilum* strain MWH-Mo1, which has a heliorhodopsin (*heR*), a DNA photolyase (*phrB*), and a CryB-type cryptochrome (*cryB*).

Several *R. lacicola* gene products were previously identified as either capable or potentially capable of light sensing: the actinorhodopsin, encoded by *actR* (*rhola_00012080*), *phrB* (*rhola_00007890*), *cryB* (*rhola_00013030*), and *cpf2* (*rhola_00011000*), encoding a putative DNA photolyase, cryptochrome, and cryptochrome-photolyase family protein, respectively (Maresca et al., 2019). In *R. lacicola*, *actR* belongs to a group of genes whose transcript abundance is high early in the light period, decreases after 1 h in the light, and increases throughout the dark period (Figure 9). The DNA photolyase and CPF2 have the same transcript abundance pattern, and are in the same expression group (Figure 9A and Supplementary Table 3). The *cryB* transcript abundance increases slightly throughout the light period, and decreases during the dark (Figure 9A), but this pattern does not cluster with any other genes. Notably, transcription of all of the putative light sensors in both strains increases immediately after the light turns on, and also increases within an hour of the light turning off.

The homologs to these potential light sensors in *A. photophilum* include *phrB*, *cryB*, and *heR*, a putative heliorhodopsin (Pushkarev et al., 2018; Maresca et al., 2019), encoded by *AURMO_01673, AURMO_00962*, and *AURMO_01564*, respectively. Transcript abundance of the putative heliorhodopsin in *A. photophilum* is fairly stable throughout the light period, then increases in the dark (Figure 9B). Transcription of the *cryB* and *phrB* homologs decreases through the light period and increases early in the dark period, then decreases at 6 h in the dark (Figure 9B).

The products of *phrB*, *cryB*, and *cpf2* are all either known or predicted to be flavoproteins (Kobayashi et al., 1989; Geisselbrecht et al., 2012; Maresca et al., 2019). The riboflavin biosynthetic genes *ribAB*, *ribD, ribH*, and *ribE* (García-Angulo, 2017) are organized into clusters in both the *R. lacicola* and *A. photophilum* genomes (*rhola_0011600-rhola_0011630* and *AURMO_00338-AURMO_*00341, respectively). In *R. lacicola*, *ribAB*, *ribD*, and *ribE* fall into expression group 3J, and although *ribH* is not in that group, it shares the same expression pattern: transcript abundance increases between t_0_ and 15 min in the light, then decreases until 1 h in the dark, then increases again (Supplementary Figure 3A). This group also includes a number of genes involved in amino acid and carbohydrate metabolic processes. The riboflavin biosynthetic genes in *A. photophilum* are in expression groups 4E and 4J and have a very similar expression pattern: increasing for 1 h in the light, then decreasing until the light turns off, then a transient increase (Supplementary Figure 3B). Expression group 4E also includes the putative DNA photolyase *phrB*, which would require the flavin cofactors synthesized by this pathway (Supplementary Table 4).

Carotenoid compounds also absorb visible light. Expression of carotenoid biosynthetic genes in *A. photophilum* decreases in the light and increases in the dark, though there is considerable variability in their expression patterns (Supplementary Figure 4B). In *R. lacicola*, the putative phytoene synthase (*Rhola_010860*) increases early in the light period, then decreases until the light turns off, then increases again. In contrast, a cluster of three genes encoding a phytoene desaturase and two putative lycopene cyclases decreases in the light, increases in the dark, and a second cluster with a second putative phytoene destaturase and a prenyltransferase increases early in the light period, then decreases for the rest of the experiment, with a transient increase early in the dark period (Supplementary Figure 4A).

## Discussion

We hypothesized that *R. lacicola* and *A. photophilum* would have similar responses to light, because they belong to the same family (Microbacteriaceae) and their genomes are broadly similar. We had also expected that light would stimulate or repress expression of specific genes, and that the effect would decay over time. Instead, we found that different pathways respond to light in the two strains, and that light and darkness alter transcription differently in both strains.

An important difference between the two strains is in energy-conserving pathways: the TCA cycle and the F_1_F_0_ ATP synthase. In *R. lacicola*, expression of the genes of the TCA cycle and the ATP synthase clearly increases in the light and decreases in response to darkness. Further, the expression patterns of carbohydrate metabolism genes suggest that *R. lacicola* may prioritize early steps of carbohydrate metabolism (glycolysis) in the dark and the TCA cycle as well as synthesis of carbohydrates and storage molecules such as starch in the light. In *A. photophilum*, there are three different expression patterns among the TCA cycle genes, but only one (*AURMO_01262*, encoding a class II fumarate hydratase) was included in an expression group (Supplementary Table 4), and transcription of genes encoding the ATP synthase appears to increase in response to both light and darkness (Figure 7). Greater expression of the TCA cycle genes in the light would likely contribute to the faster growth observed in the light in *R. lacicola*; the fact that *A. photophilum* has the same phenotype without the same gene expression patterns suggests that a different mechanism might underlie its increased growth rate in the light.

An intriguing difference between the two strains is the expression of genes encoding a putative *tad/flp* pilus and other putative competence genes. Both strains have two putative operons encoding components of the pilus, as well as the *com* system for competence. In *A. photophilum*, these genes are not coordinately expressed; in *R. lacicola*, expression of all of them clearly increases in response to darkness (Figure 8). The ways that genetic repair and exchange by these two species are regulated seem to be so different that they may have very different mechanisms for environmental adaptation and diversification. Rapid microdiversification in related freshwater Actinobacteria has been hypothesized previously (Mehrshad et al., 2018), and consistent, daily uptake and incorporation of exogenous DNA could provide a mechanism for these rapid changes in genetic makeup.

Some interesting similarities between the two strains occur in expression of potential light-sensitive proteins and the genes with similar patterns. Expression of the rhodopsin in both strains increases greatly in the dark, and the heliorhodopsin (*heR*) in *A. photophilum* has a similar expression pattern to the carotenoid biosynthetic genes. In *R. lacicola*, *actR* expression is similar to expression of about half of the carotenoid biosynthetic genes; the different expression patterns observed in this pathway suggests that different carotenoid products may be produced at different times.

The protein that senses light and signals the cells to change their activity has not yet been identified, though based on the growth rate data, it would likely be a blue-light sensing protein (Maresca et al., 2019). A new type of blue light sensor was recently identified in *Leptospirillum* (Xu et al., 2021), but no homologs to this gene were found in the genomes of *R. lacicola* or *A. photophilum*. We previously hypothesized that the putative CryB-type cryptochrome both strains have could be the light sensor (Maresca et al., 2019). Here, we observed that in *R. lacicola*, transcription of *cryB* increases during the light period, increases transiently when the light turns off, then decreases during the rest of the dark period. In contrast, transcription of *cryB* in *A. photophilum* appears to decrease through the light period, then increase greatly at the beginning of the dark period. If the CryB homologs in *R. lacicola* and *A. photophilum* regulate the light response, this difference in expression could explain why the transcriptional responses in the two strains have such different dynamics.

The transcription dynamics observed here raise questions about circadian rhythms. We previously observed that both *R. lacicola* and *A. photophilum* lack homologs of the core clock proteins (KaiA, KaiB, and KaiC) that control circadian rhythms in cyanobacteria, and that the only predicted proteins with homology to light-sensing domains such as PAS, GAF, and BLUF domains are the photolyases and cryptochromes (Maresca et al., 2019). The genomes likewise lack homologs to YtvA, a putative blue-light sensor, and the histidine kinase *KinC*, both of which have PAS domains and are expressed with circadian patterns in *Bacillus subtilis* (Eelderink-Chen et al., 2021). Although it is tempting to speculate about circadian rhythms in freshwater bacteria that inhabit surface waters and would therefore consistently be exposed to sunlight, evidence of circadian rhythms would have to come from observing an entrained pattern of activity in the absence of the light signal (Sartor et al., 2019). The oxidation state of peroxiredoxin activity has been suggested to be an indicator of circadian cycles in eukaryotes, bacteria, and archaea (Edgar et al., 2012). Both strains studied here have two peroxiredoxins in their genomes. Transcription of both peroxiredoxins in *A. photophilum* increases from 1 h after the light turns on until the light turns off, then drops sharply. In contrast, in *R. lacicola*, the peroxiredoxins have inverse transcription patterns, with one increasing early in the light period and the other decreasing, then reversal of those trends. Although we did not quantify peroxiredoxin oxidation state, peroxiredoxin transcription in both strains appears to vary in coordination with light availability. This may reflect changes in oxidative stress in the light – other genes in this category have similar transcriptional profiles – but is intriguing, given the consistent association of peroxiredoxin activity with circadian rhythms (Edgar et al., 2012).

We observed that in *R. lacicola*, light stimulates or represses expression of most light-responsive genes, and that effect decays over time, leading to curved gene expression profiles with different maxima or minima. The relatively small number of dark-responsive genes, however, suggest that to this strain, darkness is not just an absence of light, but a different stimulus altogether. We see this even more strongly in *A. photophilum*, where both light and darkness induce transient changes in gene expression. It is possible here, as in *Rhodobacter sphaeroides*, a light sensitive protein is a master regulator, and any change in light availability disrupts expression of a variety of genes (Frühwirth et al., 2012). Regardless, it suggests that *R. lacicola* and *A. photophilum* may have different signal transduction pathways and/or regulatory proteins that respond to light and darkness. Since they clearly have different networks of light- and dark-responsive genes, this is not surprising. These data lay the groundwork for experiments testing the effects of light on physiological and biochemical properties of freshwater Actinobacteria, and identifying the cellular activities that correspond to light-induced transcriptional changes in these organisms.

## Data Availability Statement

The datasets presented in this study can be found in online at the NCBI sequencing read archive under the Bioproject Numbers PRJNA454999–PRJNA455026 for *R. lacicola* and PRJNA500876–PRJNA500902 for *A. photophilum*. R scripts for all bioinformatic analyses are available at Github (https://github.com/MarescaLab/Transcriptome-Analysis).

## Author Contributions

JM designed the experiments, supervised the laboratory work and data analysis, and led the writing effort. JK did the laboratory experiments and contributed to writing and editing. PH did the bioinformatic analysis and contributed to writing. All authors contributed to the article and approved the submitted version.

## Conflict of Interest

The authors declare that the research was conducted in the absence of any commercial or financial relationships that could be construed as a potential conflict of interest.

## Publisher’s Note

All claims expressed in this article are solely those of the authors and do not necessarily represent those of their affiliated organizations, or those of the publisher, the editors and the reviewers. Any product that may be evaluated in this article, or claim that may be made by its manufacturer, is not guaranteed or endorsed by the publisher.
