# Supplementary material for: RNA-Seq Reveals that Light and Darkness Are Different Stimuli in Freshwater Heterotrophic Actinobacteria
Source: Front Microbiol. 2021 Nov 1;12:739005. doi: 10.3389/fmicb.2021.739005 (PMC8591293; doi:10.3389/fmicb.2021.739005)

SUPPLEMENTAL FIGURES

Figure 1. Representation of GO-slim terms in the genomes of *R. lacicola* and *Aurantimicrobium* sp. strain MWH-Mo1. All terms except transposition are present at similar proportions in the two genomes.

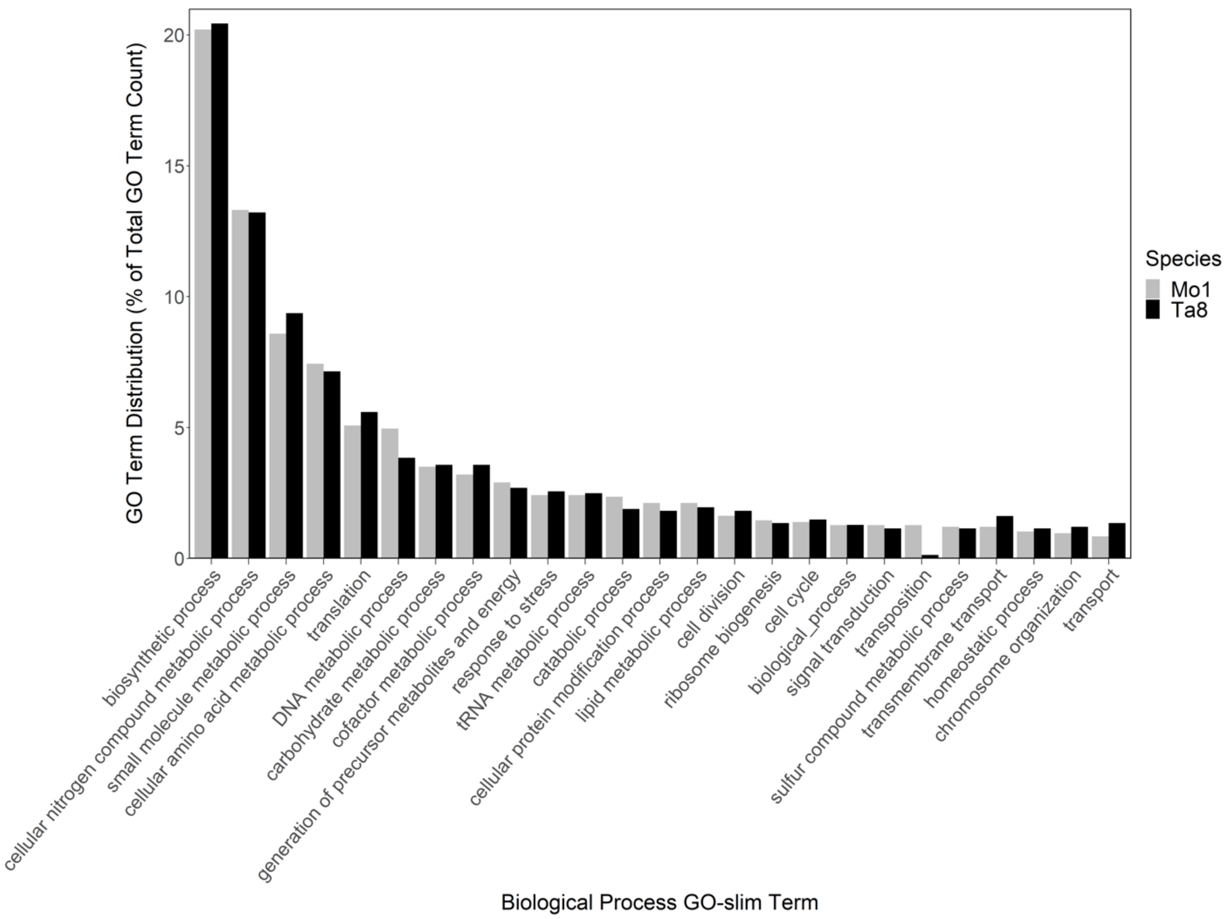

Supplemental Figure 2. Expression of peroxiredoxin genes. Expression is plotted as z-score of each transcript. (A) Putative peroxiredoxins in *R. lacicola*. (B) Putative peroxiredoxins in *Aurantimicrobium* sp. strain MWH-Mo1.

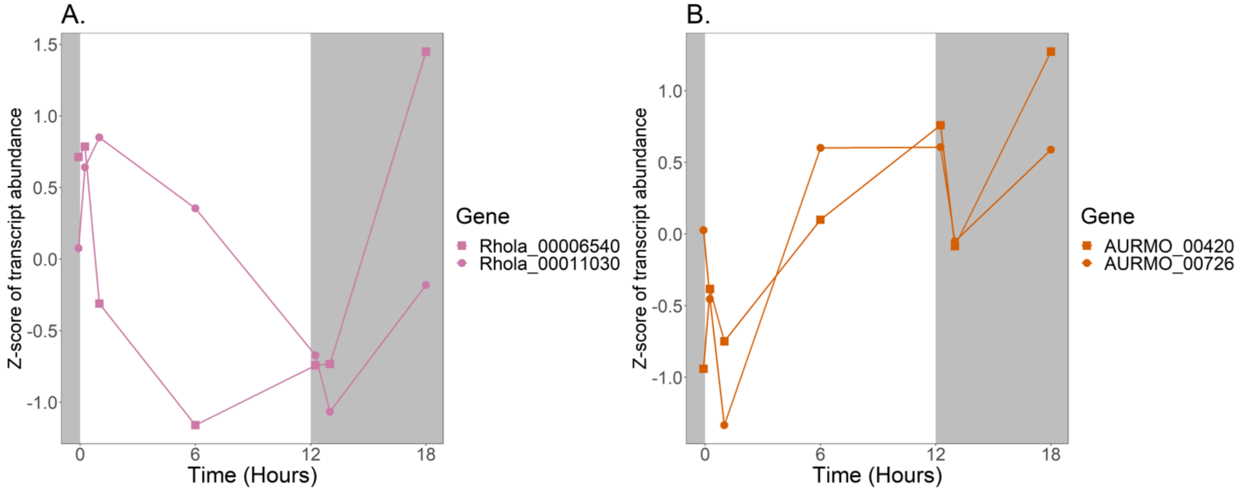

Supplemental Figure 3. Expression of putative riboflavin biosynthesis genes. In both strains, *ribH, ribAB, ribE*, and *ribD* are organized in a cluster. Expression is plotted as z-score of each transcript. (A) Putative riboflavin biosynthesis genes in *R. lacicola*. (B) Putative riboflavin biosynthesis genes in *Aurantimicrobium* sp. strain MWH-Mo1.

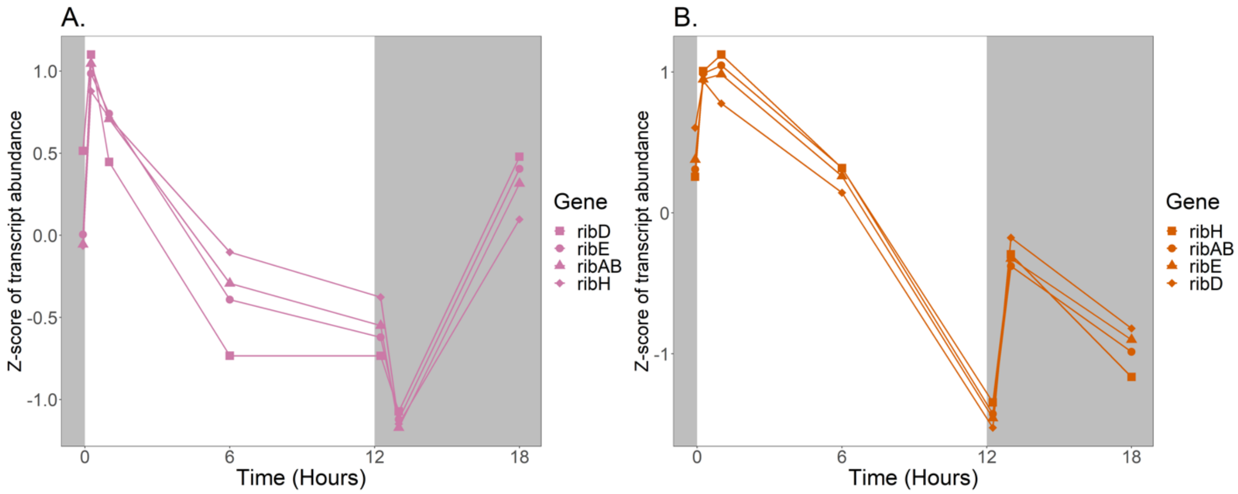

Supplemental Figure 4. Expression of carotenoid biosynthesis genes. Expression is plotted as z-score of each transcript. (A) Putative carotenoid biosynthesis genes in *R. lacicola*. (B) Putative carotenoid biosynthesis genes in *Aurantimicrobium* sp. strain MWH-Mo1.

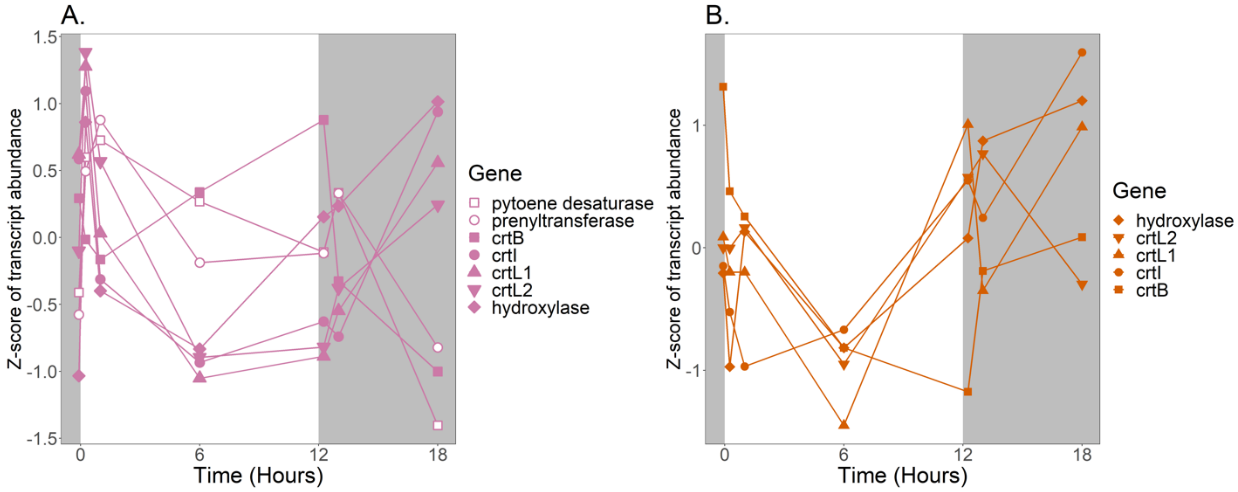

Supplement: Supplementary file 1 [file Data_Sheet_1.PDF]
